# Expanding the toolbox: another auxotrophic marker for targeted gene integrations in *Trichoderma reesei*

**DOI:** 10.1186/s40694-021-00116-5

**Published:** 2021-09-14

**Authors:** Paul Primerano, Melani Juric, Robert Mach, Astrid Mach-Aigner, Christian Derntl

**Affiliations:** Institute of Chemical, Environmental and Bioscience Engineering, TU Wien, Gumpendorfer Strasse 1a, 1060 Wien, Austria

**Keywords:** *Trichoderma reesei*, Histidine auxotrophy, ATP phosphoribosyltransferase, Marker recycling, Gene targeting, Heterologous expression

## Abstract

**Background:**

The filamentous ascomycete *Trichoderma reesei* is used for the industrial production of cellulases and holds the promise for heterologous gene expression due to its outstandingly high protein secretion rates and its long-term application in industry and science. A prerequisite for successful heterologous gene expression is the ability to insert a corresponding expression cassette at suitable loci in the genome of *T. reesei*.

**Results:**

In this study, we test and demonstrate the applicability of the *his1* gene [encoding for the ATP phosphoribosyltransferase (EC 2.4.2.17), part of the histidine biosynthesis pathway] and locus for targeted gene insertion. Deletion of the *his1* promoter and a part of the coding region leads to histidine auxotrophy. Reestablishment of the *his1* locus restores prototrophy. We designed a matching plasmid that allows integration of an expression cassette at the *his1* locus. This is demonstrated by the usage of the reporter EYFP (enhanced yellow fluorescence protein). Further, we describe a minimal effort and seamless marker recycling method. Finally, we test the influence of the integration site on the gene expression by comparing three strains bearing the same EYFP expression construct at different loci.

**Conclusion:**

With the establishment of *his1* as integration locus and auxotrophic marker, we could expand the toolbox for strain design in *T. reesei*. This facilitates future strain constructions with the aim of heterologous gene expression.

**Supplementary Information:**

The online version contains supplementary material available at 10.1186/s40694-021-00116-5.

## Background

The filamentous ascomycete *Trichoderma reesei* (teleomorph *Hypocrea jecorina* [1]) is used for the industrial production of cellulases and xylanases and has established itself as model organism for several aspects of fungal biology including regulation of gene expression, protein secretion, sexual development, and light response [2–7]. *Trichoderma reesei* has been in the focus of basic and applied research for several decades [8, 9] and holds a great promise for heterologous protein expression and secretion due to its outstandingly high protein secretion rate [2, 5]. A fundamental prerequisite for controlled heterologous protein expression is the ability to insert genes at defined loci. In a previous study, we developed a strategy for targeted gene insertions using auxotrophic markers in *T. reesei* [10]. In that study, we demonstrate that the upstream regions of the *pyr4* gene [encoding for the orotidine 5′-phosphate decarboxylase (EC 4.1.1.23)] and the *asl1* gene [encoding for the argininosuccinate lyase (EC 4.3.2.1)] as target sites for gene insertions. In a first step the promoters and the complete or partial coding regions of the genes are deleted, leading to uridine and arginine auxotrophy, respectively. The resulting strains can be used as recipient strains for gene integrations; a gene of interest is inserted upstream of the promoter regions together with the previously deleted genomic sequences. Please refer to our previous study for a detailed description of this strategy [10]. This yields strains that are isogenic to the original parent strain except for the inserted gene. Prototrophy is simultaneously re-established and can be used for selection of the gene insertion.

In this study, we describe the applicability of the *his1* gene [TRIREDRAFT_80820, encoding for the ATP phosphoribosyltransferase (EC 2.4.2.17)] as a suitable insertion locus and auxotrophic marker for gene integrations in *T. reesei*. Additionally, we test if and how the choice of the integration site effects the expression of the inserted gene. To this end, we determine the expression of the reporter EYFP (enhanced yellow fluorescence protein) in strains carrying the *eyfp* gene at the *pyr4*, the *asl1*, or the *his1* locus by comparative transcript analysis and fluorescence measurements. Additionally, we describe a minimal effort and seamless marker recycling strategy, and we construct a triple auxotrophic strain, which can be used for future multiple gene insertions.

## Results

### Deletion of *his1* leads to histidine auxotrophy in *T. reesei*

**Fig. 1:**
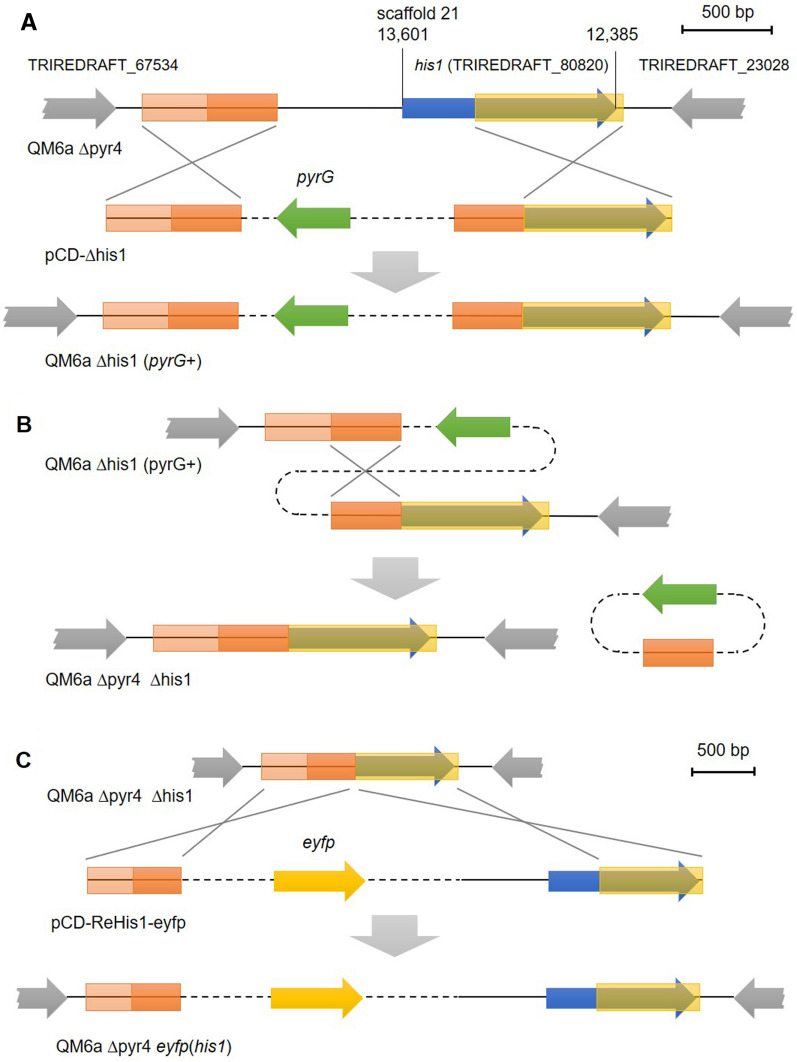
Modification of the *his1* locus during strain generations. **A** In the uridine auxotrophic recipient strain *T. reesei* QM6a Δpyr4, the *his1* gene (blue arrow) is located in close vicinity to two other genes (grey arrows; TRIREDRAFT_67534 is a predicted protein kinase, TRIREDRAFT_23028 is a hypothetical Ca2  +  permeable channel). After transformation of the plasmid pCD-Δhis1, homologous recombination may occur at the two flanks (orange and yellow boxes) resulting in the replacement of the *his1* promoter and a part of the coding sequence with the *A. fumigatus pyrG* marker (green arrow), which restores uridine prototrophy. This yields the strain *T. reesei* QM6a Δhis1 (*pyrG*  +). **B** Due to the direct repeat of a part of the 5′flank (dark orange box) in front of the 3′flank (yellow box) in the strain *T. reesei* QM6a Δhis1 (*pyrG*  +) an internal homologous recombination may occur spontaneously, which leads to the loss of the *pyrG* gene. This results in uridine auxotrophy and the generation of the double-auxotrophic strain *T. reesei* QM6a Δpyr4 Δhis1. **C** Transformation of the plasmid pCD-ReHis-eyfp into the strain *T. reesei* QM6a Δpyr4 Δhis1 may lead to a homologous recombination at the 5′ and 3′flanks (orange and yellow boxes). As the plasmid contains the previously deleted *his1* promoter and partial coding region, the native *his1* locus is restored and additionally an EYFP expression cassette integrated upstream of the *his1* promoter, yielding the strain QM6a Δpyr4 *eyfp*(*his1*)

**Fig. 2:**
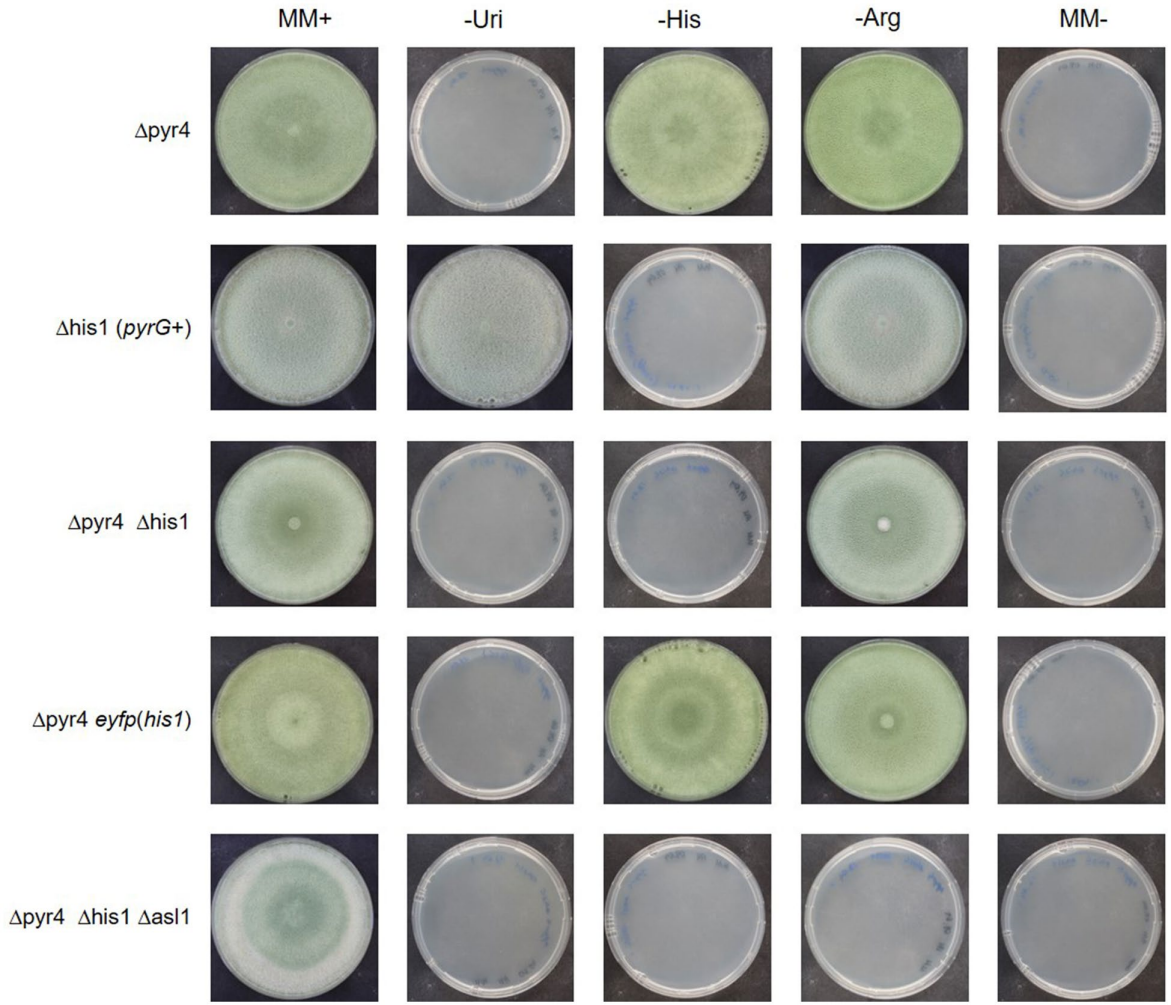
Phenotype characterization of the auxotrophic strains. The indicated strains were cultivated on minimal medium plates supplemented with 5 mM uridine, 2.5 mM arginine, and 4 mM histidine (MM  +), and on comparable plates lacking one of the three supplements (-Uri, -His, -Arg), and on minimal plates without any supplements (MM-)

First, we deleted a part of the *his1* coding region and the native promoter using a homologous recombination strategy and the *pyrG* marker (from *Aspergillus fumigatus*) (Fig. 1A). To this end, the plasmid pCD-Δhis1 was linearized and transformed into *T. reesei* QM6a Δpyr4. The correct integration was verified by PCR analyses (Additional file 1: Figure S1). The resulting strain, *T. reesei* QM6a Δhis1(*pyrG*  +) was histidine auxotroph and prototroph for uridine because the *A. fumigatus pyrG* complemented the *pyr4* deletion (Fig. 2). Notably, the deletion of *his1* also lead to a reduced growth rate on supplemented minimal and malt extract medium, delayed the onset of conidiation, and reduced the total amount of spores (not shown).

The deletion cassette contained a partial, direct repeat of the 5′flank in front of the 3′flank (Fig. 1A). The duplication of this approx. 400 bp long sequence may lead to an internal homologous recombination event, which results in a loss of the previously integrated *pyrG* gene (Fig. 1B). This event may occur randomly without an external stimulus. We selected for cells in which this internal homologous recombination event happened by cultivation the *T. reesei* QM6a Δhis1(*pyrG * +) strain on a plate containing 5-FOA (Additional File 2). This strategy enables seamless marker recycling because no genetic traces of the initially integrated *pyrG* remain at the locus (Fig. 1B). The loss of the *pyrG* gene was verified by a PCR assay (Additional File 1: Figure S1). As expected, the resulting strain, *T. reesei* QM6a Δpyr4 Δhis1 was auxotrophic for histidine and uridine (Fig. 2) and grew slower and exhibited delayed and reduced conidiation compared to the *T. reesei* QM6a Δpyr4 (not shown).

### Targeted gene insertion at the *his1* locus

**Fig. 3:**
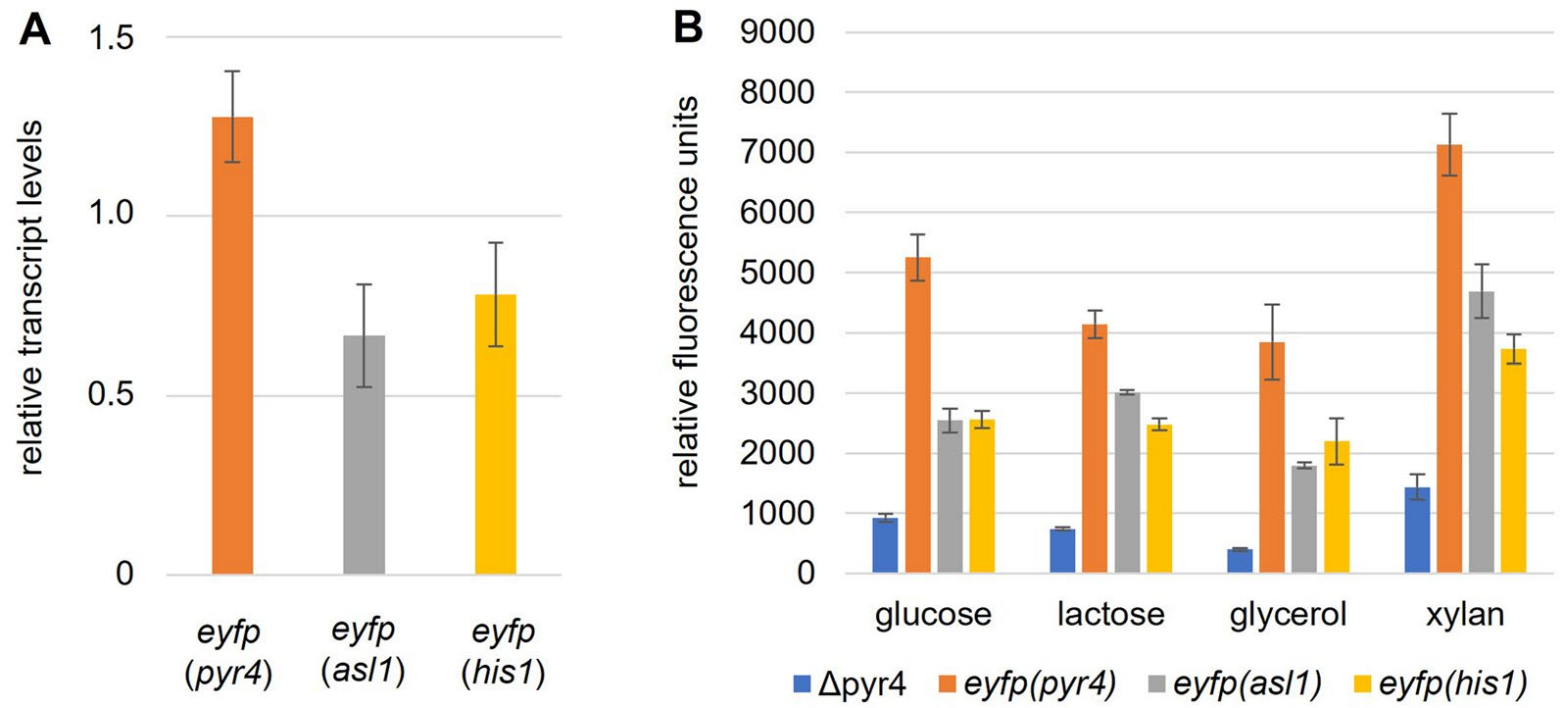
Expression analysis of EYFP on transcript and enzyme level. **A** The EYFP reporter strains (Table 1) carrying the expression cassette at either the *pyr4*, the *asl1*, or the *his1* locus were cultivated in a 12-well plate in 1.5 ml MAM containing glucose, lactose, glycerol, or xylan as carbon source. After incubation at 30 °C for 48 h, RNA was extracted, and cDNA was synthesized. The relative transcript levels of the *eyfp* were determined in a RT-qPCR assay using *act1* and *sar1* for normalization and the Pfaffl method [15] for calculation. QM6a *eyfp*(*pyr4*) on glucose was used as reference sample. The arithmetic average of all samples from all carbon sources are depicted in the bar chart. Error bars represent standard deviation. **B** The strains *T. reesei* QM6a Δpyr4, and the EYFP reporter strains carrying the expression cassette at either the *pyr4*, the *asl1*, or the *his1* locus were cultivated in a fluorescence 96-well plate in MAM containing glucose, lactose, glycerol, or xylan as carbon sources. After incubation at 30 °C without agitation for 72 h, the total fluorescence (ex 490, em 510–570) was measured. Bars represent the arithmetic average of three independent replicates. Error bars represent standard deviation

Next, we tested, whether we can use the *his1* locus as insertion site for a targeted gene integration, and whether we could use the *his1* genes as selection marker for the transformation. To this end, we transformed the linearized plasmid pCD-ReHis1-eyfp into *T. reesei* QM6a Δpyr4 Δhis1 (Fig. 1C) and selected for the reestablishment of histidine prototrophy. The correct integration was verified by PCR analyses (Additional file 1: Figure S2). The resulting strain *T. reesei* QM6a Δpyr4 *eyfp*(*his1*) was still auxotrophic for uridine but had regained prototrophy for histidine (Fig. 2) and was expressing EYFP (Fig. 3). The reinsertion of *his1* also restored normal growth rate and conidiation behavior like in the parent strain *T. reesei* QM6a Δpyr4 on minimal and malt extract medium (not shown).

### Effects of integration site on gene expression

In a previous study, we constructed two other EYFP expression strains analogously to *T. reesei* QM6a Δpyr4 *eyfp*(*his1*), namely QM6a *eyfp*(*pyr4*) and QM6a *eyfp*(*asl1*). These three strains carry the very same *eyfp* expression cassette at the *his1*, the *pyr4* and the *asl1* locus, respectively [10]. Notably, each strain bears only a single copy of the *eyfp* gene (Additional File 3; [10]). Next, we tested if and how of the insertion locus effects the gene expression of the EYFP marker. To this end, we cultivated the three latter strains together with *T. reesei* QM6a Δpyr4 on different carbon sources and measured the transcript levels of *eyfp* (Fig. 3A) and the EYFP fluorescence (Fig. 3B). As the experimental setup does not allow determining the biomass, the fluorescence units could not be normalized to the acquired biomass. However, the strains grew equally fast on the used carbon sources in a parallel cultivation in clear well plates (Additional File 4). Consequently, the fluorescence values of the different strains grown on the same carbon sources can be compared. We observed approx. two-fold higher *eyfp* transcript levels (Fig. 3A) and significantly higher fluorescence (Fig. 3B) in the strain carrying the EYFP expression cassette at the *pyr4* locus compared to the other two strains, which were similar to each other (Fig. 3B; Additional File 5). This demonstrates that all three loci can be used for heterologous gene expression and that the choice of the integration locus influences the gene expression.

### Construction of a triple auxotrophic recipient strain

Next, we decided to construct a recipient strain for multiple gene insertions for future studies and applications. To this end, we transformed the linearized plasmid pCD-Δasl1 [10] into *T. reesei* QM6a Δpyr4 Δhis1 and selected for hygromycin resistance, because the deletion cassette contains the corresponding resistance gene (Additional File 1: Figure S3). Please refer also to [10] for a detailed description and depiction of the *asl1* deletion strategy. The deletion of the *asl1* promoter and part of the coding region, was confirmed by a suitable PCR analysis (Additional File 1: Figure S3). The resulting strain *T. reesei* QM6a Δpyr4 Δhis1 Δasl1 was auxotrophic for uridine, histidine, and arginine (Fig. 2) and may be used as recipient strain in the future.

## Discussion

In this study, we demonstrated that the *his1* locus can be used as integration site for gene expression cassettes and that the *his1* gene can be used as auxotrophic marker in *T. reesei*. We observed that the deletion of the *his1* promoter and a part of the coding region leads to histidine auxotrophy, but also negatively affected the growth rate and conidiation. We speculate that this might be a result of the connection of the histidine and purine biosynthesis pathways [11]. During one reaction of the histidine biosynthesis pathway, AICAR (5-Aminoimidazole-4-carboxamide-1-beta-D-ribofuranosyl 5′-Monophosphate) is formed as co-product (https://www.genome.jp/entry/R04558). AICAR is an important intermediate for the biosynthesis of purines and is involved in other biological processes [11]. It appears, that AICAR cannot be provided in sufficient amounts through other metabolic pathways in the *his1* deletion strains. However, the re-establishment of the *his1* locus re-instates prototrophy and normal growth and sporulation behavior. This needs to be considered for the design of a strain construction strategy; the final strain must contain a functional *his1* locus.

In a previous study, we described the applicability of two other genes, *pyr4* and *asl1* for targeted gene insertions [10]. We routinely use the therein described strains and the markers in our research group, because no expensive or toxic antibiotics are needed, and the resulting strain do not carry additional marker genes, which might interfere with the planned gene expression. In this study, we demonstrated that the choice of the integration locus has a strong influence on the gene expression. This should also be considered for the strain design. The combination of differently strong promoters with different integration sites may facilitate fine-tuning of the final gene expression rate. This is of course highly speculative and should be tested in further studies.

When comparing the EYFP expression in the three *eyfp* bearing strains, we observed a strong influence of the different carbon sources on the fluorescence, but not on transcript levels (small standard deviation in the transcript analysis). This seems contradictory at first glance but can be explained by the different growth rates of *T. reesei* on the tested carbon sources. We speculate that *eyfp* is transcribed at a constant rate regardless of the carbon source, but the different growth rates on the different carbon sources lead to different amounts of acquired biomass which in turn produces and accumulates more or less EYFP. A normalization to the biomass would probably solve this problem, but the performed experiment did not allow determining the biomass in the fluorescence well plates.

Further, we described a minimal effort and seamless marker recycling method, that relies on an internal homologous recombination between two direct repeats of a natural genomic sequence (Fig. 1B). This is a random and spontaneous process that may occur during the normal cell cycle of *T. reesei*. It is also interesting to speculate how and if CRISPR-mediated genome editing may be combined with the here presented minimal effort and seamless marker recycling method. If a suitable recognition site for the Cas9 enzymes is generated by the internal homologous recombination, CRISPR may be used to open the target site for enhanced transformation and integration efficiency.

## Conclusions

We could demonstrate the applicability of the *his1* gene for targeted gene integration and as an auxotrophic marker in *T. reesei*, which expands the toolbox for future applications of this fungus as host for heterologous gene expression. We further demonstrated the applicability of a minimal effort and seamless marker recycling system, which will facilitate future strain construction efforts, because several genomic manipulations may be performed without the need of several marker genes and/or expensive and toxic compounds.

## Methods

### Fungal strains and cultivation conditions

All *T. reesei* strains (Table 1) used in this study were maintained on malt extract agar at 30 °C. Uridine, Arginine, Histidine, 5-FOA, and Hygromycin B were added when applicable to a final concentration of 5 mM, 2.5 mM, 4 mM, 1 mg/ml, and 113 U/ml, respectively.

**Table 1:** *T. reesei* strains used in this study

Designation	Description	Source
QM6a Δpyr4	Wild-type-like strain with deficiency of the non-homologous end joining repair pathway, and uridine auxotrophy	[10]
QM6a Δhis1 (*pyrG* +)	Histidine auxotrophic strain, obtained through transformation of QM6a Δpyr4 with pCD-Δhis1, carries the *A. fumigatus pyrG* gene	This study
QM6a Δpyr4 Δhis1	Uridine and histidine auxotrophic strain, obtained through *pyrG* excision in QM6a Δhis1 (pyrG +)	This study
QM6a Δpyr4 eyfp (his1)	EYFP-reporter strain, expression cassette integrated at this *his1* locus, obtained through transformation of QM6a Δpyr4 Δhis1 with pCD-ReHis-eyfp	This study
QM6a eyfp (pyr4)	EYFP-reporter strain, expression cassette integrated at this *pyr4* locus	[10]
QM6a eyfp (asl1)	EYFP-reporter strain, expression cassette integrated at this *asl1* locus	[10]
QM6a Δpyr4 Δhis1 Δasl1	Uridine, histidine, and arginine auxotrophic strain, obtained through transformation of QM6a Δpyr4 Δhis1 with pCD-Δasl1, recipient strain for multiple gene integrations	This study

For cultivations, *T. reesei* was grown in Mandels-Andreotti medium (MAM) (8.9 g/L Na_2_HPO4∙2 H_2_O, 1.4 g/L (NH_4_)_2_SO_4_, 2 g/L KH_2_PO_4_, 0.3 g/L MgSO_4_, 0.4 g/L CaCl_2_, 0.3 g/L urea, 1 g/L peptone, 20 mL/L trace elements (5 mg/L FeSO_4_∙7 H_2_O, 1.6 mg/L MnSO_4_∙H_2_O, 1.4 mg/L ZnSO_4_∙H_2_O and 2 mg/L CoCl_2_∙2 H_2_O), pH adjusted to 5 with citric acid) [12] containing 1% (w/v) of the respective carbon source. Culture were either grown in 20 ml in Erlenmeyer flasks in a rotary shaker at 30 °C and 180 rpm, or in 100 µl in fluorescence 96-well plates (sterile, flat bottom, black) at 30 °C without agitation. A total of 10^9^ conidia per liter (final concentration) was used as the inoculum in both cases.

### Auxotrophy testing

For auxotrophy testing, 5 µl of a 10^7^ spores/ml suspension were applied to the middle of a minimal medium plate with or without supplements. As minimal medium, MAM without peptone and glucose as carbon source was used. Plates were incubated at 30 °C for 1 week.

### Plasmid constructions

PCRs for cloning purposes were performed with Q5 High-Fidelity DNA Polymerase (New England Biolabs (NEB), Ipswich, MA, USA) according to the manufacturer’s instructions. All used primers are listed in Table 2. PCR products were cloned into *Eco*RV-digested pJET1.2 (Thermo Fisher Scientific Inc., Waltham, MA, USA) and verified by sequencing (at Microsynth, Balgach, Switzerland). The fragments were released for subsequent cloning purposes by digestion with suitable restriction endonucleases (NEB).

**Table 2:** Primers used in this study

Number	Name	Sequence (5′–3′)
A70	80820_5fwd-BspEI	TCCGGACTCTCAACCATGGCTTCAGAC
A71	80820_5rev-EcoRI	GAATTCTTATGGTTTGGTACTAGGTACTG
A72	80820_5fwd2-AflII	CTTAAGAGATGAAGTACTGCTATAAGCGC
A73	80820_5rev-SOE	CTTGACCTGATCCTTATGGTTTGGTACTAGGTACTG
A74	80820_3fwd-SOE	CCAAACCATAAGGATCAGGTCAAGGAGCACGATG
A75	80820_3rev-NsiI	ATGCATCTTTTCAGCACGGCACTTAC
A272	80820_5rev-MCS	GCTAGCTACCTTAGGCTGGAATTCCTGGGATCCTTATGGTTTGGTACTAGGTACTG
A273	P80820_fwd-MCS	GCTAGCCGTCATATGGGTCTGCAGTAGCACTGGACTTGATCACAG
A274	80820_3rev-ClaI	ATCGATCTTTTCAGCACGGCACTTAC
A294	Ppki_fwd-BamHI	GGATCCGACGGCCAGTGAATTCTCG
220	Tcbh2_rev_NheI	GCTAGCGCTATTAACGTTTGGAAAGC
A99	80820_5fwd2	GTTCTAAAGCCTCGTCGAGAG
A100	80820_3rev2	CCTTCAGCGTGAACGGACTAG
A63	pyrG_3fwd	ACATTGTGCCTGTCATTAAACG
A62	pyrG_5rev	AATGGGGTAGACAGGCAGAAC
233	Ppki_5rev	CAGCAGCCACGACAAAGC
45	cbh2terMF	CATCACAACCTCGTCTCCCTC
336	Ppki_Mrev	ATTAGGTGATGCTGCGCG
A772	sar1fw	TGGATCGTCAACTGGTTCTACGA
A773	sar1rev	GCATGTGTAGCAACGTGGTCTTT
A774	act1f	TGAGAGCGGTGGTATCCACG
A775	act1r	GGTACCACCAGACATGACAATGTTG
B069	eYFP_fwd_qPCR	TACAACTACAACAGCCACAACG
B070	eYFP_rev_qPCR	TTACTTGTACAGCTCGTCCATG
C12	cbh1_fwd_qPCR	GGCTAAAAGTACATAAGTTAATGCC
C13	cbh1_rev_qPCR	GACTTACATTTCAATATGGACCACT
212	Ppki_fwd-Kpn2I	TCCGGACGGCCAGTGAATTCTCGAG
214	Tcbh2_rev_PstI	CTGCAGGCCATCCAAAGAGCTCAACC

For the construction of pCD-Δhis1, the 5′ flank was amplified by PCR using the primers 80820_5fwd-BspEI and 80820_5rev-EcoRI and chromosomal DNA of *T. reesei* QM6a Δpyr4 and inserted into pJET-pyrG [13]. Next, the partial direct repeat of the 5′flank was fused to the 3′flank by a splicing by overlap extension PCR. The fragments were amplified using the primers 80820_5fwd2-AflII and 80820_5rev-SOE or 80820_3fwd-SOE and 80820_3rev-NsiI. The fusion PCR fragment was inserted into the latter plasmid yielding pCD-Δhis1 (Fig. 1A; Additional File 6).

For the construction of pCD-ReHis1, the 5′flank of *his1* was amplified with the primers 80820_5fwd-BspEI and 80820_5rev-MCS and inserted into pJET1.2 (Thermo Fisher Scientific) in the opposite direction of the *eco47IR* gene. Next the promoter and the coding region of *his1* was amplified with the primers P80820_fwd-MCS and 80820_3rev-ClaI and inserted into the latter plasmid via *Nhe*I and *Cla*I. The resulting plasmid pCD-ReHis1 (Additional File 7) contains a multiple cloning site (*Bam*HI, *Eco*RI, *Nhe*I, *Nde*I, *Pst*I) between the 5’flank and the promoter of *his1* to facilitate insertion of further genes.

For the construction of pCD-ReHis1-eyfp (Additional File 8), the expression cassette for EYFP, containing the constitutive *pki* promoter, a codon-optimized *eyfp* gene, and the *cbh2* terminator, was amplified with the primers Ppki_fwd-BamHI and Tcbh2_rev_NheI using pCD-EYFP [10] as template and inserted into pCD-ReHis1 via *Bam*HI and *Nhe*I.

For the construction of the standard plasmids for the qPCR assay to determine the copy number of *eyfp*, a part of the *cbh1* coding region and the *eyfp* expression cassette were amplified using the primers cbh1_fwd_qPCR and cbh1_rev_qPCR, and Ppki_fwd-Kpn2I and Tcbh2_rev_PstI, and chromosomal DNA of QM6a Δpyr4 and pCD-EYFP [10] as template, respectively, and inserted into pJET1.2 (Thermo Fisher Scientific).

### Fungal transformations

The protoplast generation and polyethylene glycol mediated transformation of *T. reesei* was performed as described previously [14]. Typically, 15 µg of linearized plasmid DNA (digested with *Not*I, precipitated with ethanol, resuspended in 15 µl sterile ddH_2_O) was used for the transformation of 10^7^ protoplasts (in 100 µl). Selection was described previously [10]. Resulting candidates were subjected to homokaryon purification by streaking conidia on plates with selection medium containing 0.1% (w/v) Igepal CA-630 (Sigma-Aldrich, part of Merck KGaA, Darmstadt, Germany).

### Marker recycling

For the minimal effort marker recycling, the strain *T. reesei* QM6a Δhis1(*pyrG*  +) was incubated on MAM plates without peptone containing uridine, histidine, and 5-FOA. The plate was incubated at 30 °C for up to 4 weeks, until the *pyrG* marker was lost due to a random internal homologous recombination (Fig. 1B) and the fungus gained 5-FOA tolerance (Additional File 2).

### Isolation of chromosomal DNA

Chromosomal DNA was isolated from mycelium by grinding in liquid nitrogen followed by a phenol/chloroform extraction [14]. RNA was degraded using RNaseA (Thermo Fisher Scientific). DNA was precipitated with isopropanol, washed with 70% ethanol, and dissolved in ddH_2_O.

### Genotype testing by PCR

For testing the genotype, 10 ng of chromosomal DNA were used as template in a 25-µl-PCR using OneTaq polymerase (NEB) according to the manufacturer’s instructions. All used primers are listed in Table 2. For the agarose gel electrophoresis of the amplification products the 1 kb Plus DNA Ladder (NEB) was used as standard.

### Determination of the *eyfp* copy number

Dilutions of the chromosomal DNA of the EYFP-expressing strains were used as template in a qPCR assay targeting the *eyfp* and the *cbh1* gene. For comparison, plasmids carrying the target size were used. The relative copy number of *eyfp* in relation to *cbh1* was calculated (Additional File 3) using the Pfaffl method [15].

### RNA extraction

Fungal strains were cultivated in Erlenmeyer flasks for 48 h, mycelia and supernatants were separated by filtration through Miracloth (Merck Millipore, part of Merck KGaA, Darmstadt, Germany). Approx. 0.05 g of harvested mycelia were resuspended in 1 ml RNAzol RT (Sigma-Aldrich) and lyzed using a Fast-Prep-24 (MP Biomedicals, Santa Ana, CA, USA) with 0.37 g of small glass beads (0.1 mm diameter), 0.24 g of medium glass beads (1 mm diameter), and a single large glass bead (5 mm diameter) at 6 m/s for 30 s. Samples were incubated at room temperature for 5 min and then centrifuged at 12,000*g* for 5 min. 750 µl of the supernatant were mixed with 750 µl ethanol and RNA isolated using the Direct-zol RNA Miniprep Kit (Zymo Research, Irvine, CA, USA) according to the manufacturer’s instructions. This Kit includes a DNAse treatment step. The concentration and purity were measured using the NanoDrop ONE (Thermo Scientific).

### Transcript analysis by RT-qPCR

500 ng of isolated total RNA was reverse transcribed using the LunaScript RT SuperMix (NEB) according to the manufacturer’s instructions. The resulting cDNA was diluted 1:50 and 2 µl were used as template in a 20 µl reaction using the Luna Universal qPCR Master Mix (NEB) according to the manufacturer’s instructions. All reactions were performed in technical duplicates on a Rotor-Gene Q system (Qiagen, Hilden, Germany). Calculations of the relative transcript levels were performed according to the Pfaffl method [15] using the reference genes *sar1* and *act1* for normalization according to [16].

### Fluorescence measurements

The strains were cultivated in fluorescence 96 well plates in technical triplicates in two independent experiments, and in parallel in technical triplicates in a transparent 96 well plates to determine the optical density. After 72 h cultivation the total fluorescence or the optical density of the cultures was measured in a Glomax Multi Detection System (Promega, Madison, WI, USA) using the blue filter kit (excitation peak wavelength at 490 nm, emission wavelengths between 510 and 570 nm) or absorbance at 600 nm.

## Supplementary Information

**Additional file 1: **Genotype verification of the constructed strains.

**Additional file 2: **Marker recycling due to a spontaneous internal recombination leading to the loss of the *pyrG* gene.

**Additional file 3: **Determination of the copy number of the integrated *eyfp* gene.

**Additional file 4: **Growth curves of the *T. reesei *strains QM6a Δpyr4, Δpyr4 eyfp (his1), eyfp (pyr4), and eyfp (asl1) on different carbon sources in a 96-well plate.

**Additional file 5: **Raw data and calculation for the fluorescence units of the *T. reesei *strains QM6a Δpyr4, Δpyr4 eyfp (his1), eyfp (pyr4), and eyfp (asl1) on different carbon sources in a 96-well plate.

**Additional file 6: **Genomic sequence of pCD-Δhis1.

**Additional file 7: **Genomic sequence of pCD-ReHis1.

**Additional file 8: **Genomic sequence of pCD-ReHis-eyfp.

## Data Availability

All data and materials described are freely available for scientific and academic purposes upon request to the corresponding author.

## Abbreviations

5-FOA: 5-Fluororotic acid
AICAR: 5-Aminoimidazole-4-carboxamide-1-beta-D-ribofuranosyl 5′-Monophosphate
EYFP: Enhanced yellow fluorescence protein
MAM: Mandels-Andreotti medium
PCR: Polymerase chain reaction
RT-qPCR: Quantitative reverse transcription PCR
