# Supplementary material for: Expanding the toolbox: another auxotrophic marker for targeted gene integrations in Trichoderma reesei
Source: Fungal Biol Biotechnol. 2021 Sep 14;8:9. doi: 10.1186/s40694-021-00116-5 (PMC8442374; doi:10.1186/s40694-021-00116-5)
Supplement: Supplementary file 1 — Additional file 1: Genotype verification of the constructed strains. [file 40694_2021_116_MOESM1_ESM.pdf]

Expanding the toolbox: another auxotrophic marker for targeted gene integrations in *Trichoderma reesei*

Paul Primerano[1], Melani Juric[1], Robert Mach[1], Astrid Mach-Aigner[1], Christian Derntl[1§]

[1] Institute of Chemical, Environmental and Bioscience Engineering, TU Wien, Gumpendorfer Strasse 1a, 1060 Wien, Austria

[§] address correspondence to christian.derntl@tuwien.ac.at

Additional File 1 - Genotype verification of the constructed strains

Figure S1 Genotype testing of QM6a Δhis1 (*pyrG*+) and QM6a Δpyr4 Δhis1

Figure S2 Genotype testing of QM6a Δpyr4 *eyfp* (*his1*)

Figure S3 Genotype testing of QM6a Δpyr4 Δhis1 Δasl1

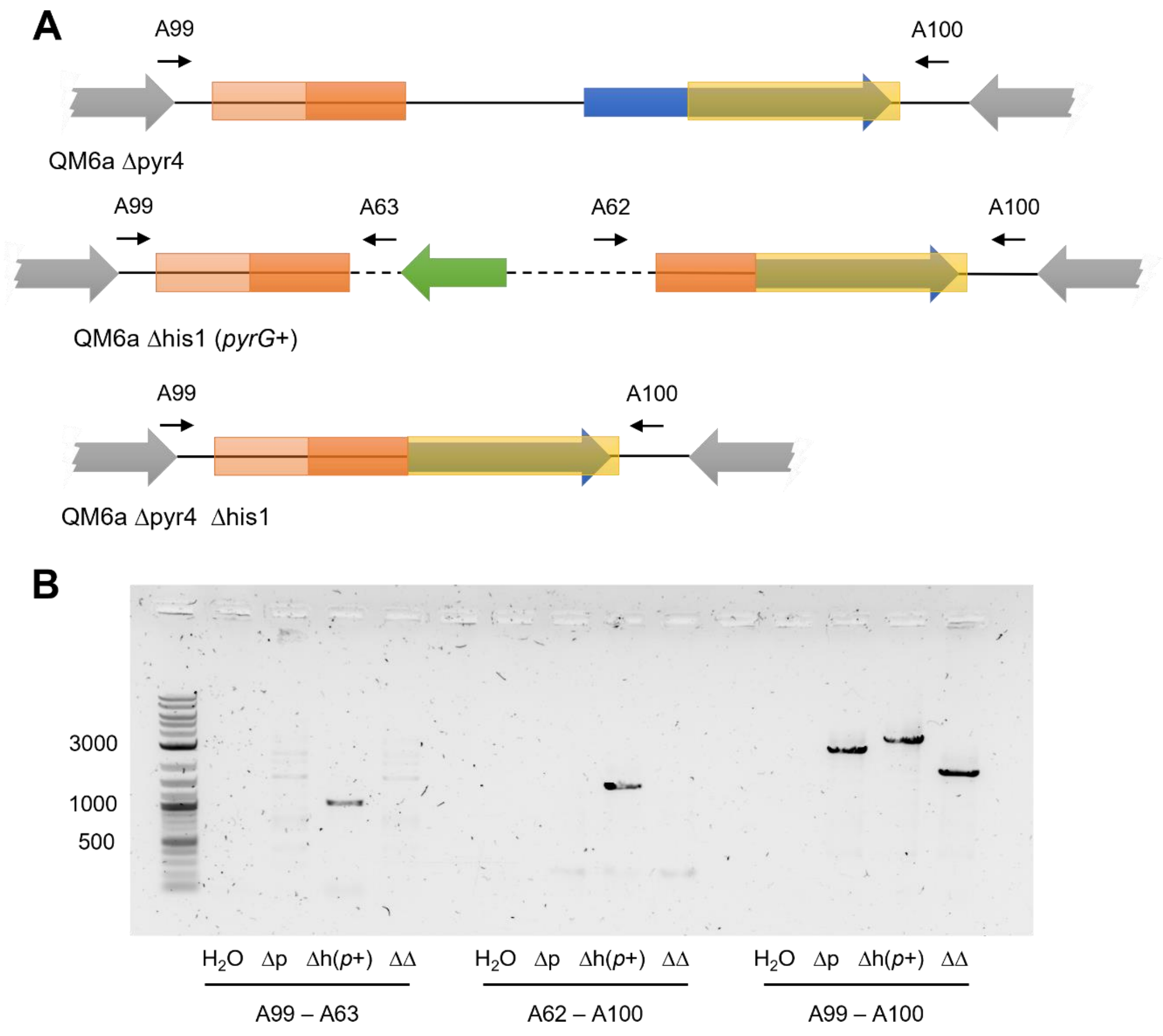

Figure S1 Genotype testing of QM6a Δhis1 (*pyrG*+) and QM6a Δpyr4 Δhis1. Analytical PCR assays were performed targeting the *his1* locus (**A**) in the strains QM6a Δpyr4 (Δp), QM6a Δhis1 (*pyrG*+) (Δ(*p+)*, and QM6a Δpyr4 Δhis1 (ΔΔ) using the indicated primers (sequences given in Table 2) and chromosomal DNA of the strains as template. Sterile water (H_2O) was used instead of a template as negative control for each PCR assay. PCR results were visualized on an agarose gel (**B**) using the 1 kb Plus DNA Ladder (NEB) as standard. Expected sizes for the PCR reactions: A99 – A63, 1042 bp; A62 – A100, 1473 bp; A99 – A100, 3082 bp for Δp, 3783 bp for (Δ(p+), 1968 bp for ΔΔ.

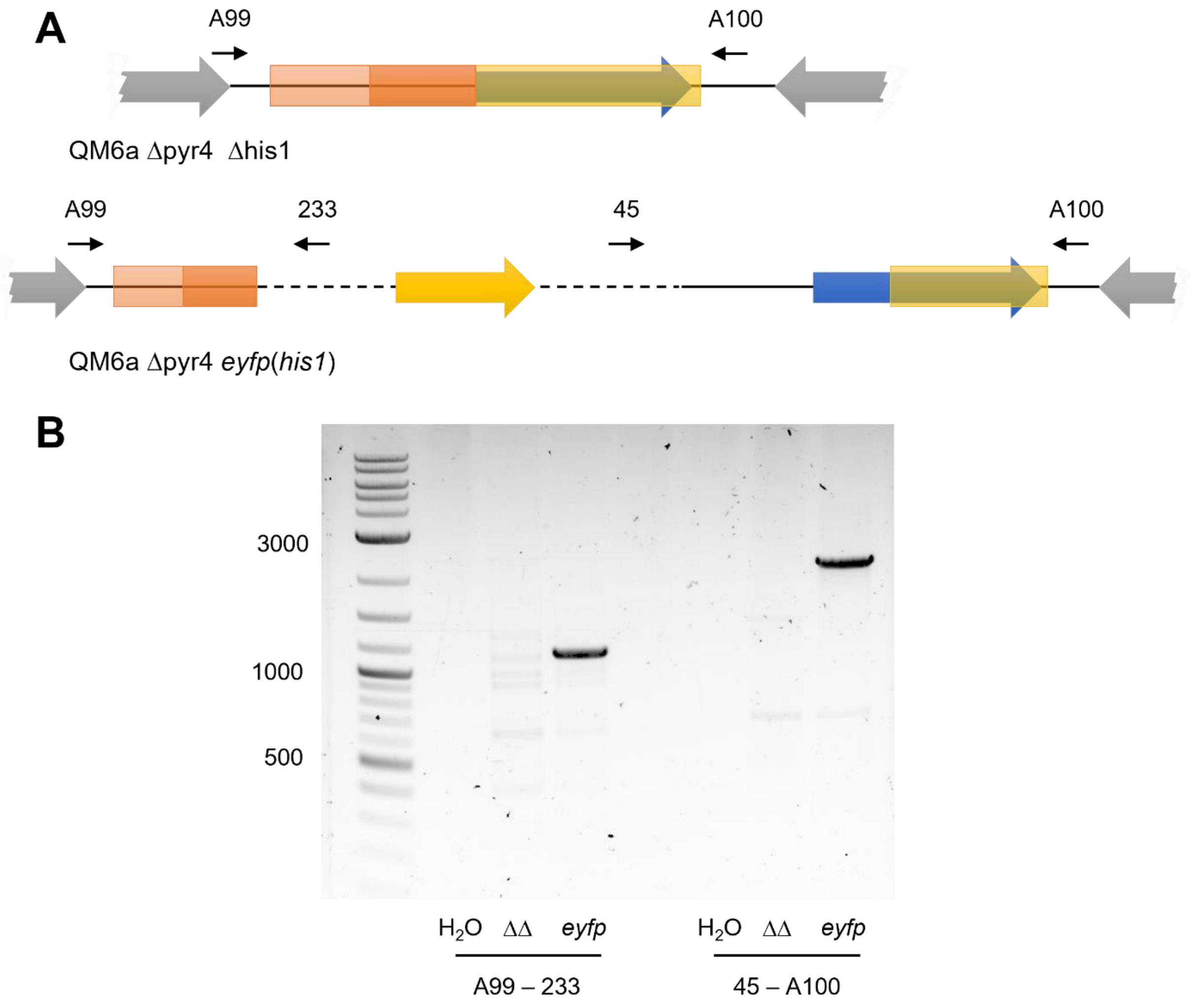

Figure S2 Genotype testing of QM6a Δpyr4 *eyfp* (*his1*). Analytical PCR assays were performed targeting the *his1* locus (**A**) in the strains QM6a Δpyr4 Δhis1 (ΔΔ) and QM6a Δpyr4 *eyfp* (*his1*) (eyfp) using the indicated primers (sequences given in Table 2) and chromosomal DNA of the strains as template. Sterile water (H_2O) was used instead of a template as negative control for each PCR assay. PCR results were visualized on an agarose gel (**B**) using the 1 kb Plus DNA Ladder (NEB) as standard. Expected sizes for the PCR reactions: A99 – 233: 1139 bp; 45 – A100: 2250 bp.

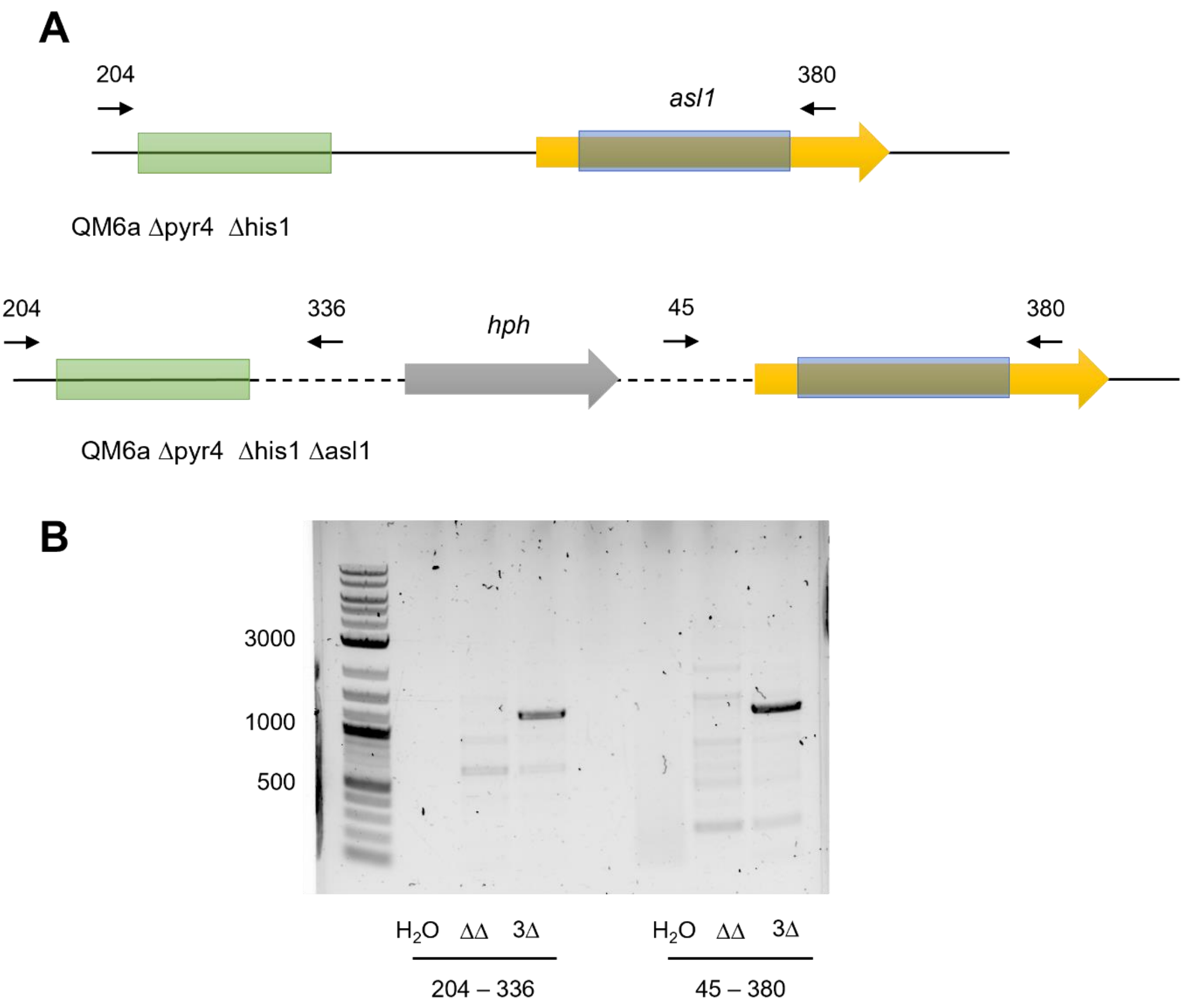

Figure S3 Genotype testing of QM6a Δpyr4 Δhis1 Δasl1. Analytical PCR assays were performed targeting the *asl1* locus (**A**) in the strains QM6a Δpyr4 Δhis1 (ΔΔ) and QM6a Δpyr4 Δhis1 Δasl1 (3Δ) using the indicated primers (sequences given in Table 2) and chromosomal DNA of the strains as template. Sterile water (H_2O) was used instead of a template as negative control for each PCR assay. PCR results were visualized on an agarose gel (**B**) using the 1 kb Plus DNA Ladder (NEB) as standard. Expected sizes for the PCR reactions: 204 – 336: 1242 bp; 45 – 380: 1208 bp.
