# Supplementary material for: Expanding the toolbox: another auxotrophic marker for targeted gene integrations in Trichoderma reesei
Source: Fungal Biol Biotechnol. 2021 Sep 14;8:9. doi: 10.1186/s40694-021-00116-5 (PMC8442374; doi:10.1186/s40694-021-00116-5)
Supplement: Supplementary file 2 — Additional file 2: Marker recycling due to a spontaneous internal recombination leading to the loss of the pyrG gene. [file 40694_2021_116_MOESM2_ESM.pdf]

Expanding the toolbox: another auxotrophic marker for targeted gene integrations in *Trichoderma reesei*

Paul Primerano[1], Melani Juric[1], Robert Mach[1], Astrid Mach-Aigner[1], Christian Derntl[1§]

[1] Institute of Chemical, Environmental and Bioscience Engineering, TU Wien, Gumpendorfer Strasse 1a, 1060 Wien, Austria

[§] address correspondence to christian.derntl@tuwien.ac.at

Additional File 2 - Marker recycling due to a spontaneous internal recombination leading to the loss of the *pyrG* gene.

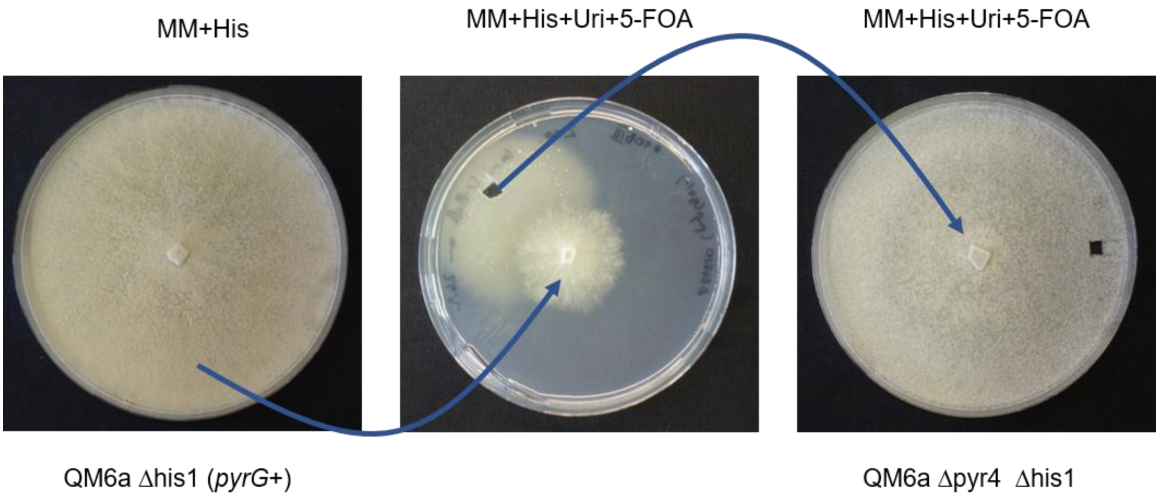

The strain *T. reesei* QM6a Δhis1(*pyrG*+) was incubated on MAM plates without peptone (MM) containing uridine, histidine, and 5-FOA. The plate was incubated at 30°C for up to 4 weeks, until the *pyrG* marker was lost due to a random internal homologous recombination (Fig. 1B) and the fungus gained 5-FOA tolerance.
